# The association between perceived bedtime autonomy, sleep patterns, and daytime functioning in adolescents

**DOI:** 10.3389/frsle.2026.1719668

**Published:** 2026-05-25

**Authors:** Sarah Hartley, Sylvie Royant-Parola, Sylvain Dagneaux, Frédérique Aussert, Catherine Tobie, Sandrine Launois, Marta Fernandez-Bolanos, Amandine Rey, Stéphanie Mazza

**Affiliations:** 1 Réseau Morphée, Garches, France; 2 Physiology Department, Sleep Unit, AP-HP Raymond Poincaré Hospital, Versailles-St Quentin en Yvelines University, Garches, France; 3 CNRS, INSERM, Centre de Recherche en Neurosciences de Lyon, Université Claude Bernard Lyon 1, Bron, France

**Keywords:** adolescent, screen use, sleep autonomy, sleep duration, social jetlag

## Abstract

**Introduction:**

Sleep duration in adolescents is determined by bedtime as weekday getting up times are fixed by school starting times. The impact of adolescents' choice of bedtime (bedtime autonomy) during the week and at the weekend on sleep duration and sleep timing has been little studied.

**Methods:**

A cross sectional questionnaire survey of adolescents responding to an online survey recorded sleep habits on school nights and weekends, screen use and daytime repercussions. Bedtime autonomy (determining one's own bedtime) on weeknights and at weekends, sleep deprivation (< 7 h in bed/night), school night sleep restriction (≥2 h difference in time in bed on school nights vs. weekends), social jetlag (midpoint of time in bed variation >2 h between school nights and weekends), difficulty waking, level of anxiety/depression hospital anxiety and depression scale (HAD), sleepiness (FSSA-8 sleepiness scale), duration of screen use and timing of screen use (evening, after bedtime, during the night) were determined.

**Results:**

Two thousand eight hundred ninety-five questionnaires were returned of which 2,512 were completed by adolescents (70% female, mean age 14.46 ± 2.08). Fourteen percent were non autonomous (NA), 21% were autonomous at the weekend only weekend autonomy (WA) and 65% at the weekend and on weeknights total autonomy (TA). Autonomy increased with age (*p* < 0.001) and female sex (*p* < 0.001). Weekend autonomy is granted to younger children than weeknight autonomy (13.61 ± 2.0 vs. 16.4 ± 2.8): nearly all participants were totally autonomous by the age of 18. In a model adjusting for age, sex, mood, and sleepiness potential sleep deprivation was linked to WA and more strongly to TA while social jetlag was only associated with WA.

**Conclusion:**

Bedtime autonomy is an important factor in determining bed time duration and sleep rhythms in adolescents.

## Highlights

- Bedtime autonomy is linked to age and leads to later bedtimes on weeknights.
- Total bedtime autonomy (weekend and weeknights) is strongly associated with potential sleep deprivation on weeknights but is not associated with social jetlag.
- Weekend bedtime autonomy is associated with potential sleep deprivation on weeknights and social jetlag.
- Parents and adolescents differ in their perception of autonomy, with higher estimates of bedtime autonomy from adolescents than from parents.

## Introduction

The interactions of the physiological and socio-cultural determinants of sleep in adolescents have been described as a perfect storm which conspire to limit sleep duration (Carskadon, 2011). Sleep needs for adolescents have been estimated to be approximately 9 h per night with wide interindividual variation (Hirshkowitz et al., 2015). However, in practice sleep duration is often well below 9 h and this decreases across adolescence with a reduction of about 1 h from the ages of 9–17 (Thompson et al., 2024). Daytime sleepiness and difficulties waking in the morning are frequent in adolescents, implying that sleep time is insufficient (Carskadon, 1990).

The changes underpinning the reduction in sleep duration are complex and relate to physiological changes in the underlying neuronal mechanisms of sleep and to socio-cultural factors. Both the homeostatic and circadian systems change during adolescence. The homeostatic system shows a slower build-up of sleep pressure (Jenni et al., 2005) which, coupled with a delay in circadian phase (Carskadon, 1990), leads to a tendency for later bedtimes. The physiological circadian delay is potentially exacerbated by evening screen use driven by both homework and social activities (Hartley et al., 2022). Wake up times for adolescents are fixed in the week by early school start times which, in the context of later bedtimes, lead to reduced sleep duration and accumulated fatigue during the school week (Hansen et al., 2005). Adolescents habitually sleep in at weekends to catch up for lost sleep, leading to a destabilization of circadian rhythms with a widely varying midpoint of sleep between weeknights and the weekend (de Souza and Hidalgo, 2014).

In childhood and early adolescence, parents typically set bedtimes based on both cultural influences and perceived sleep needs. Adolescence is a period of transition from childhood to adulthood with the gradual development of independence as a central psychodevelopmental stage (Lohaus, 2018). Determining when to go to bed (bedtime autonomy) is a key feature of independence (Carskadon, 2011). Bedtime autonomy may be total (weekends and school nights) or partial (weekends only). The choice of bedtime is driven not only by internal cues such as fatigue or sleepiness but also by external social and cultural activities. If internal cues and external activities are in conflict, delaying going to bed may occur (Pu et al., 2022).

Bedtime autonomy in adolescents has been relatively little studied, and the prevalence of total and partial autonomy, their evolution over adolescence and their impact on sleep and wake are unclear (Ewing et al., 2024; Maskevich et al., 2022; Meijer et al., 2016; Tashjian et al., 2019; Illingworth et al., 2025; Short et al., 2019). We hypothesized that autonomy would increase progressively over adolescence and that total autonomy would lead to reduced time in bed. The objective of this study was to explore the impact of bedtime autonomy, both on weeknights and at weekends, on time in bed, sleep regularity, daytime sleepiness and mood disorders.

## Methods

All respondents to a free anonymous online survey on adolescent sleep were included. The Réseau Morphée is a non-profit organization, funded by the French regional health authority (ARS-IDF), dedicated to improving sleep. Information and access to the questionnaire was via the website of the Réseau Morphée. All adolescents were encouraged to participate and no incentives were provided. The questionnaire was aimed at adolescents aged 11–18 who wanted to know more about their sleep. It could be filled in by parents or by adolescents with their parents. Parental consent for the use of anonymised data was obtained prior to completion of the questionnaire. The study was approved by the scientific committee of the Réseau Morphée and by the Commission Nationale Informatique et Liberté (CNIL), 8013081 19/12/2016.

Data on who completed the questionnaire (adolescent or parent) were recorded. Sleep autonomy was assessed by the question “do you decide when you go to bed” (response yes/no) for both weeknights and at the weekend. The framing of the question made it clear that the response was either the adolescents own choice sleep autonomy or a parental choice not autonomous (NA). The motivation for going to bed was also sought with multiple responses including homework, social activities, siblings, fatigue and parental encouragement.

### Sleep related measures

Two continuous variables (mean time in bed on weeknights and mean time in bed at the weekend) were calculated from bedtime and getting up times reported for the week and weekends. A binary variable, potential sleep deprivation (yes/no) was defined as a time in bed < 7 h based on the internationally recommended sleep duration for adolescents as this has been shown to have clinical impact on daytime functioning (Hirshkowitz et al., 2015). Variability in sleep was evaluated by a continuous variable measuring the difference between the time in bed midpoint in the week and at weekends. A binary variable, social jetlag (yes/no) was defined as >2 h difference between the time in bed midpoint in the week and at weekends (Wittmann et al., 2006).

Chronotype was determined by the five-item reduced Morningness–Eveningness Questionnaire (rMEQ): range 4–26, with higher scores indicating morningness. Cutoff scores were eveningness: < 12; neither: 12–17; morning: >17 (Caci et al., 2009).

Participants were asked about the amount of screen use in the evening, in bed and during the night (after lights out) and whether they put off going to bed (bedtime procrastination). Questions relating to screen time are reported in Supplementary Table 1.

### Daytime functioning

Daytime sleepiness was measured with the French sleepiness scale for adolescents FSSA-8 (Gustin et al., 2023). Participants with a score >10 were considered sleepy. The need to sleep during the day was evaluated with a yes/no answer.

Symptoms relating to mood and anxiety were evaluated with the Hospital Anxiety and depression scale (HAD) with subscales for anxiety (HAD-A) and depression (HAD-D) which has been validated in teenagers (Bocéréan and Dupret, 2014; White et al., 1999).

### Statistical analysis

Data was collated in Excel. Quantitative variables (time in bed, bedtime and getting up time, and HAD) were described by mean and standard deviation. Normality was estimated by Kolmogorov–Smirnov testing and data were analyzed by *t*-test or by non-parametric tests (Kruskall–Wallis) as appropriate. Categorical variables such as sex, evening screen use, night-time screen use and symptoms of sleep pathology were described by percentage and analyzed by Chi^2^ tests. The Bonferroni correction was applied for multiple tests with a statistical significance level of *p* = 0.0026.

**Figure 1:**
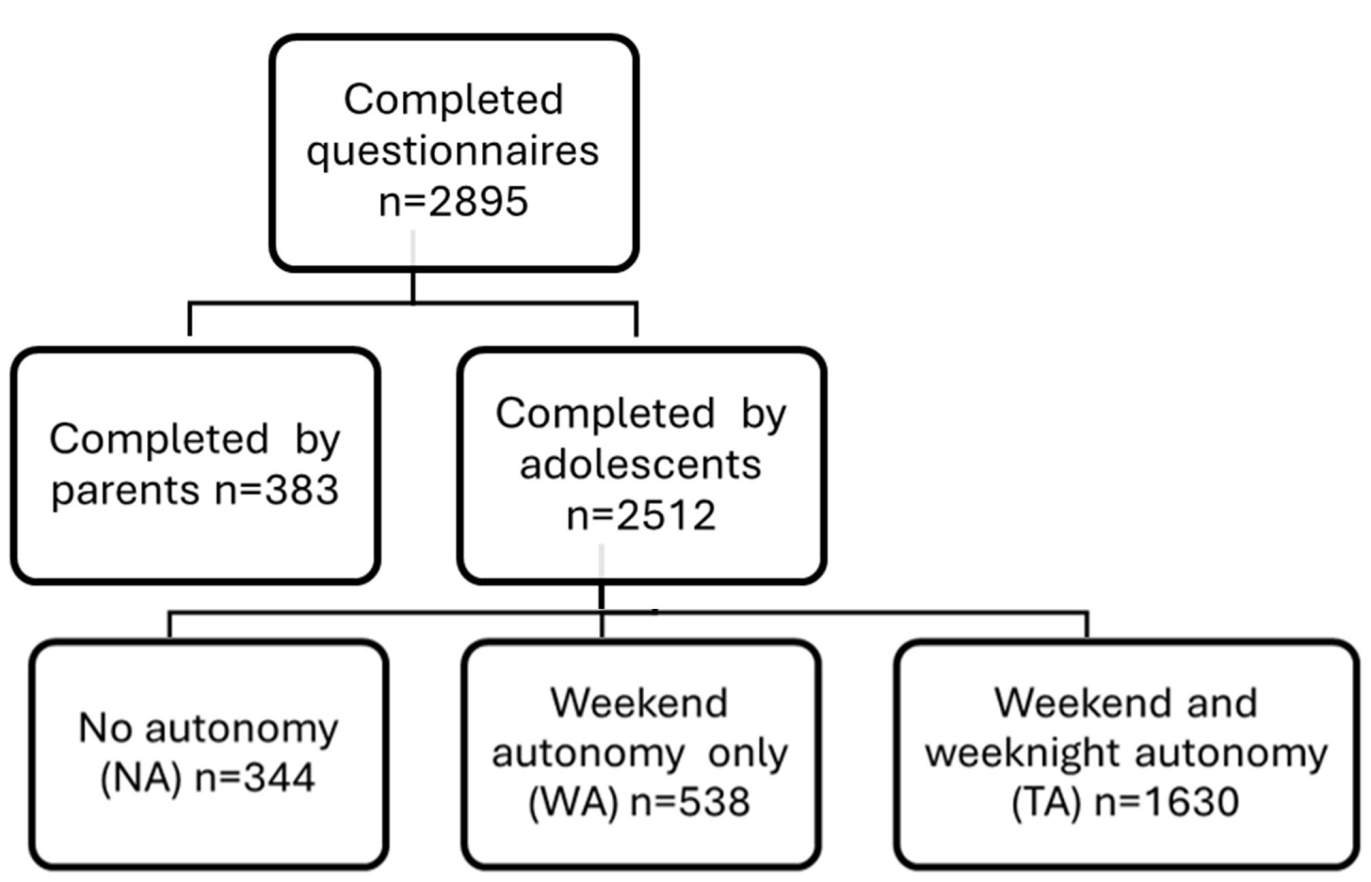
Flow chart.

The questionnaire could be completed both by parents for their adolescent and by the adolescents themselves (with parental consent). Initial analysis compared parent and adolescent assessments of autonomy. Further analysis was performed using only data from questionnaires completed by adolescents (see flow chart Figure 1). Adolescents were divided into three groups: adolescents without bedtime autonomy, (NA), adolescents with weekend autonomy only (WA) and adolescents with both weekend and weeknight autonomy (TA).

Linear regression models were used to examine whether bedtime autonomy groups (NA, WA, and TA) were associated with time in bed during the week, time in bed at the weekend, bedtime during the week, bedtime at the weekend, and the difference between midpoint time in bed at the weekend and in the week. Models were adjusted for age, sex and MEQ.

Two logistic regression models were used to examine whether the independant variables NA, WA, TA were associated with the binary dependent variables potential sleep deprivation and social jetlag. Models were adjusted for age and sex, as these are known to influence sleep duration (Hirshkowitz et al., 2015; Franco et al., 2020), symptoms of mood and anxiety, sleepiness, and chronotype (MEQ).

Missing variables were excluded from the analysis. Analysis was performed using JASP (version 0.19.1, The Netherlands).

## Results

### Participants

Two thousand eight hundred ninety-five questionnaires were fully completed. Three hundred eighty-three by parents for their child and 2,512 by adolescents (see Figure 1 for flow chart). Overall 70% of respondents were female with a mean age of 14.46 (SD = 2.08). When analyzing only questionnaires completed by adolescents (*n* = 2,512), 73% of respondents were female with a mean age of 15.5 (SD = 2.9; see Table 1).

**Table 1:** Population: sociodemographic characteristics.

Variables	Responses from adolescents	Responses from parents	Total responses
Number	2,512	383	2,895
Age mean (SD)	15.5 (2.9)	13.4 (2.4)	14.46 (2.08)
Sex (female)	73%	51%	70%
IMC	20.38 (5.38)	19.51 (4.018)	20.34 (5.185)
Not autonomous (NA)	344 (14%)	151 (39%)	495 (17%)
Autonomy at the weekend only (WA)	538 (21%)	100 (36%)	638 (22%)
Autonomy at the weekend and on weeknights (TA)	1,630 (65%)	132 (35%)	1,762 (61%)

### Sleep autonomy

Sleep autonomy was assessed during the school week and weekends. Overall, 17% of adolescents were NA, 22% were autonomous at the weekend only (WA) and 61% were totally autonomous both at the weekend and on weeknights (TA).

A significant difference was found in autonomy evaluated by adolescents and by parents. Among parents who completed the questionnaire for their adolescents, 39 vs. 14% of adolescents (*p* < 0.0001) thought that their adolescents were NA, and 35 vs. 65% of adolescents (*p* < 0.0001) thought that their adolescents were autonomous on both weekends and weeknight (TA).

#### Autonomy, age, and sex

**Figure 2:**
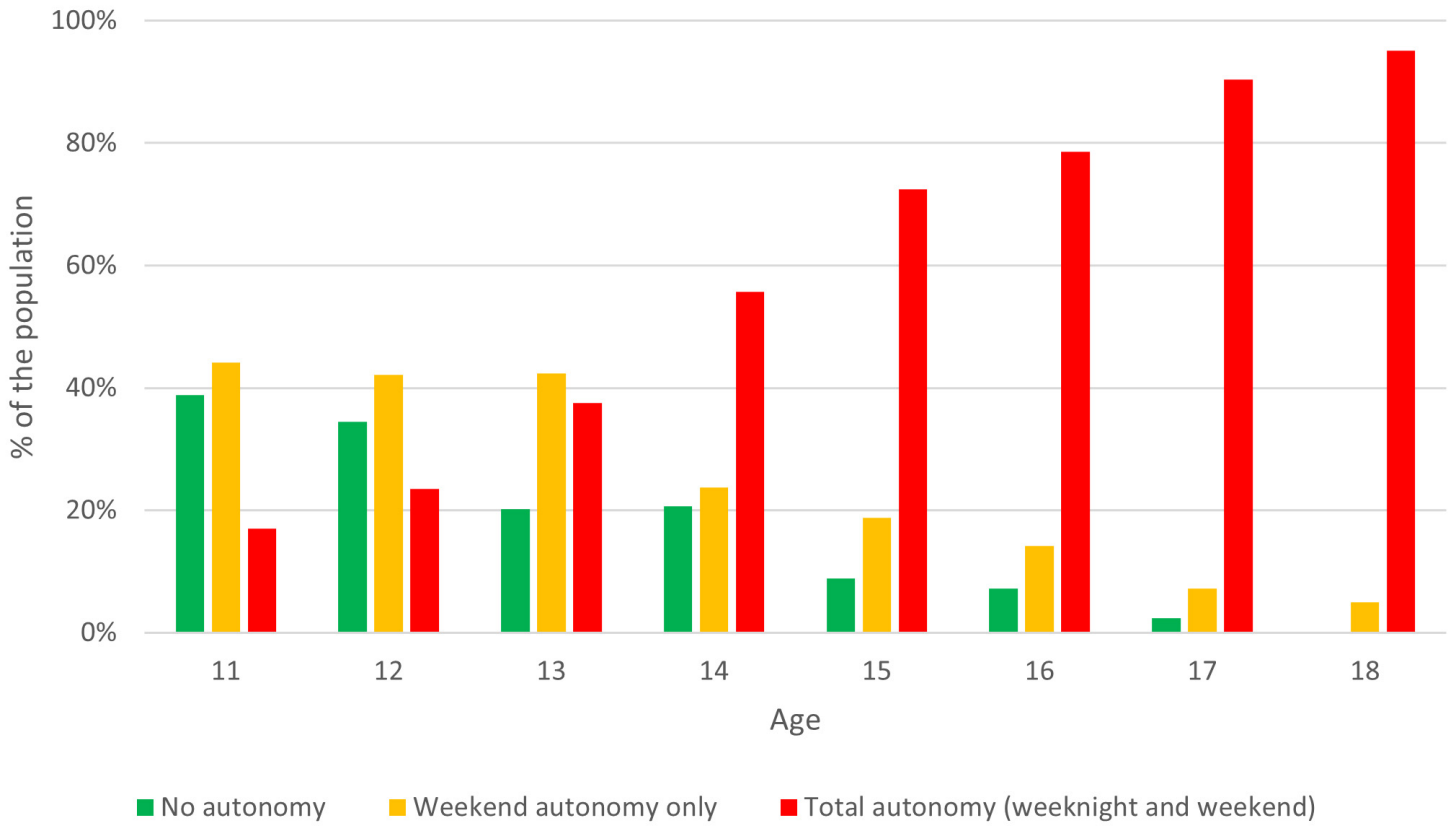
Age and bedtime autonomy (% of the population) during the week and at the weekend.

Further analysis excluded questionnaires completed by parents and incorporated only data provided by adolescents (Table 2). Autonomy was significantly correlated with age (Figure 2), with an increase in autonomy observed with advancing age: autonomy at the weekend was achieved at a younger age than autonomy during the week.

**Table 2:** Factors associated with autonomy in adolescents: bivariate analysis.

Variables	Non autonomous (NA)	Bedtime autonomy at the weekend only (WA)	Bedtime autonomy at the weekend and on weeknights (TA)	*p*
Number	344	538	1,630	
Sex (% female)	221 (64%)	398 (74%)	1,225 (75%)	< 0.001^*^
Age mean (SD, range)	13.3 (2.03 11–17)	13.61 (2.0 11–18)	16.4 (2.8 11–18)	< 0.001^**^
Sleep				
Bed time in the week mean (SD minutes)	21:33 (60)	21:53 (77)	23:11 (99)	< 0.001^**^
Getting up time in the week mean (SD minutes)	06:54 (36)	06:48 (33)	06:56 (53)	NS^**^
Bed time at the weekend mean (SD minutes)	22:48 (80)	23:34 (101)	00:34 (122)	< 0.001^**^
Getting up time in the weekend mean (SD minutes)	09:34 (88)	10:01 (100)	10:20 (109)	< 0.001^**^
Time in bed week mean (SD minutes)	9:21 (66)	08:55 (81)	07:46 (96)	< 0.001^**^
Time in bed weekend mean (SD minutes)	10:46 (85)	10:26 (102)	09:46 (108)	< 0.001^**^
Sleep deprivation: time in bed < 7 h in the week (%)	6 (2%)	27 (5%)	310 (19%)	< 0.001^*^
Social jetlag (%)	121 (35%)	268 (53%)	818 (50%)	< 0.001^*^
MEQr evening type (%)	337 (31%)	169 (32%)	587 (42%)	< 0.001^*^
Screen use				
Screen use >120 minutes in the evening (%)	37 (11%)	77 (15%)	639 (46%)	< 0.001^*^
Screen based activities once in bed (%)	36 (11%)	81 (15%)	576 (42%)	< 0.001^*^
Screen use during the night (%)	17 (5%)	23 (4%)	131 (9%)	< 0.001^*^
Bedtime procrastination (putting off going to bed) (%)	237 (67%)	389 (72%)	1,181 (73%)	NS^*^
Consequences on mood and daytime function				
Symptoms of depression HADD mean (SD)	5.57 (3.9)	5.53 (4.1)	6.69 (4.0)	< 0.001^**^
Symptoms of anxiety HADA mean (SD)	9.4 (4.4)	9.28 (4.7)	10.12 (4.8)	< 0.001^**^
Need to sleep during the day (%)	124 (36%)	215 (39%)	891 (55%)	< 0.001^*^
Daytime sleepiness FSS-8	6.2 (4.8)	6.8 (4.5)	8.9 (4.8)	< 0.001^**^

^*^Chi^2^ test.

^**^Kruskall–Wallis test.

#### Autonomy, time in bed, and sleep timing

Bivariate analysis showed that autonomy, both TA and WA, was significantly associated with later bedtimes, both in the week and at the weekend, and with later getting up times at the weekend (see Table 2). Getting up times in the week were not significantly different. Autonomy was associated with a markedly reduced time in bed on weeknights with TA adolescents spending on average 95 min less in bed compared to the NA group. Potential sleep deprivation (< 7 h in bed in the week) affected 19% of adolescents in the TA group compared to only 2% in the NA group (*p* < 0.001). Autonomy was also associated with an evening chronotype which was more marked in the TA and WA than in the NA group.

Autonomy contributed to irregular sleeping patterns: TA and WA were significantly associated with social jetlag.

**Figure 3:**
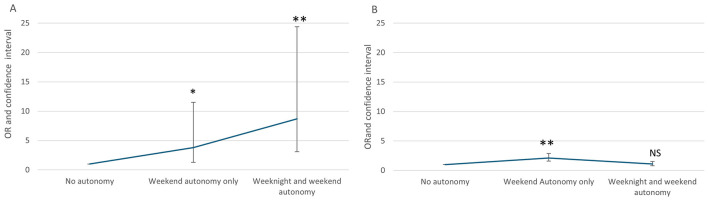
The effect of autonomy on time in bed and social jetlag. **(A)** Potential sleep deprivation (less than 7 hours in bed). **(B)** Social jetlag. *p < 0.05, **p < 0.001.

Linear regression analyses showed that, compared with the group NA, TA was associated with later bedtimes on weeknights (β 1.153, *p* < 0.0001) and shorter time in bed on weeknights (β −1.225, *p* < 0.0001) while WA showed intermediate effects (bedtime weeknight β 0.288, *p* = 0.003 time in bed weeknights β −0.378, *p* < 0.0001). Compared with the group NA, WA was associated with a higher time in bed midpoint difference weeknight vs. weekend (β 0.459, *p* < 0.0001) compared to TA (β 0.329, *p* < 0.0001; Figure 3).

#### Autonomy and screen use

TA was significantly associated with excessive screen use, defined as >120 min after dinner in the evening, screen use in bed and with screen use during the night.

#### Autonomy and symptoms of mood and anxiety

TA but not WA was significantly associated with an increase in symptoms of depression (HAD-D) and anxiety (HAD-A).

#### Autonomy and daytime sleepiness

TA, but not WA was associated with a significant increase in sleepiness expressed as a need to sleep during the day and also as measured by the FSSA-8.

#### Autonomy and factors prompting bedtime

Adolescents who were totally autonomous were prompted to go to bed principally by finishing activities which were often screen based (TA 48 vs. NA 11% *p* < 0.001) or by fatigue (TA 31 vs. NA 6% *p* < 0.001; Table 3). The group NA were typically prompted to go to bed by finishing homework (NA 75 vs. TA 7%). In all groups parental intervention to prompt bedtime was extremely low: NA 3 vs. TA 2%.

**Table 3:** Factors prompting adolescents to go to bed and bedtime autonomy.

Variables	Non autonomous (NA)	Autonomous weekends only (WA)	Autonomous week and weekend (TA)
Fatigue (%)	**22 (6%)**	**53 (10%)**	**507 (31%)**
Finished homework (%)	**259 (75%)**	**361 (67%)**	**119 (7%)**
Sibling bedtime (%)	**13 (4%)**	**30 (6%)**	**191 (12%)**
Parental prompting (%)	12 (3%)	16 (3%)	31 (2%)
Activities (screen based activities vs. non screen based activities such as reading) (%)	**38 (11%)**	**78 (15%)**	**782 (48%)**

Significant data (*p* < 0.05) is shown in bold.

### Multivariate analysis

**Figure 4:**
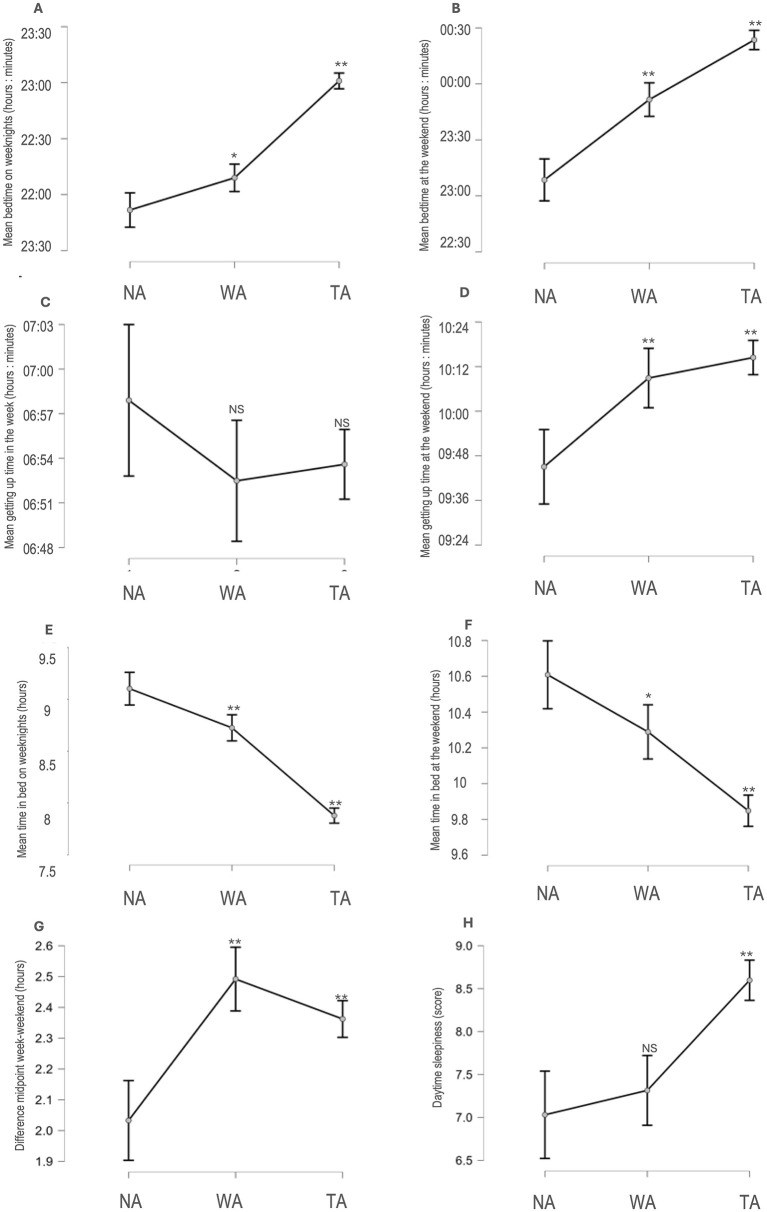
The effect of autonomy on sleep and vigilance. **(A)** Mean bedtime on weeknights, **(B)** mean bedtime at weekends, **(C)** mean getting up time in the week, **(D)** mean getting up time at the weekend, **(E)** mean time in bed on weeknights (hours), **(F)** mean time in bed on weekends (hours), **(G)** mean difference midpoint week-weekend (hours), **(H)** mean daytime sleepiness (score). *p < 0.05, **p < 0.001.

Factors associated with the independent binary variables potential sleep deprivation and social jetlag were examined in a model adjusted for age, sex, symptoms of mood and anxiety disorders, sleepiness, and MEQ (see Table 4 and Figure 4). For potential sleep deprivation the logistic regression model was statistically significant Δ*X*^2^ (2,243) 484.4, *p* < 0.0001 with a moderate relationship (McFadden *R*^2^ 0.276). Compared with the reference group NA, TA had higher odds of potential sleep deprivation (OR: 8.8, CI: 3.2–25, *p* < 0.0001) than WA (OR: 3.8, CI: 1.3–11.3, *p* = 0.019). Heavy screen use in the evenings showed higher odds (OR: 4.4, CI: 3.2–6.1, *p* < 0.0001) while the MEQ score was protective (OR: 0.8, CI: 0.77–0.84, *p* < 0.0001).

**Table 4:** Multivariant analysis: factors associated with potential sleep deprivation and social jetlag.

Variables	Potential sleep deprivation			Social jetlag		
	OR	IC 95%	*p*	OR	IC 95%	*p*
Sex (female vs. male)	1.18	0.8–1.7	0.4	0.88	0.7–1.1	0.25
Age	**1.1**	**1.01–1.2**	**0.03**	**1.1**	**1.1–1.2**	**< 0.0001**
WA: autonomy at weekends only	**3.8**	**1.3–11.3**	**0.02**	**2.1**	**1.6–2.9**	**< 0.0001**
TA: autonomy on weekends and weeknights	**8.9**	**3.2–25**	**< 0.0001**	1.1	0.8–1.5	0.5
Screen use (≥120 min) in the evening	**4.4**	**3.2–6.2**	**< 0.0001**	**1.33**	**1.1–1.67**	**0.013**
Screen use in bed	1.01	0.8–1.4	0.9	**1.4**	**1.1–1.8**	**0.002**
Screen use in the middle of the night	1.4	0.93–2.2	0.09	1.2	0.8–1.7	0.4
Symptoms of anxiety (HADA) score	1.02	0.98–1.1	0.3	**0.97**	**0.95–0.99**	**0.02**
Symptoms of depression (HADD) score	1.03	0.99–1.07	0.9	1.03	0.99–1.05	0.09
Daytime sleepiness (FSS-8) score	1.01	0.98–1.05	0.5	**1.03**	**1–1.05**	**0.01**
Morning-eveningness (MEQr) score	**0.8**	**0.77–0.84**	**< 0.0001**	**0.82**	**0.79–0.83**	**< 0.0001**

Significant data (*p* < 0.02) is shown in bold.

For social jetlag the model was statistically significant Δ*X*^2^ (2,243) 420.4, *p* < 0.0001 but with a weak relationship (McFadden *R*^2^ 0.134). WA (OR: 2.1, CI: 1.6–2.9, *p* < 0.0001) but not TA (OR: 1.1, CI: 0.8–1.5, *p* = 0.5) was associated with higher odds of social jetlag compared to the reference NA. Heavy screen use in the evenings (OR: 1.3, CI: 1.06–1.66, *p* < 0.013) and once in bed (OR: 1.4, CI: 1.1–1.8, *p* = 0.002) increased the odds while the MEQ score was protective (OR: 0.8, CI: 0.79–0.84, *p* < 0.0001).

## Discussion

This is the first study to our knowledge to examine bedtime autonomy with a detailed focus on age and the development of weekend vs. weeknight autonomy. Results showed that adolescent autonomy is an important factor in determining both time in bed and sleep regularity in adolescents.

We found a marked difference between parents' and adolescents' perceptions of bedtime autonomy: although parents reported that one-third of their children are autonomous in determining bedtime during the week, over 60% of adolescents thought this was the case. Parents are known to have an idealized view of their childrens' sleep patterns (Short et al., 2013) and this discrepancy has been found previously in studies in young adolescents (Gunn et al., 2019).

Bedtime autonomy, both during the week and at the weekend, was affected by age confirming previous studies (Tashjian et al., 2019; Carskadon, 1982). We found that weekend autonomy develops first and is followed by autonomy during the week. The majority of adolescents achieve autonomy on weeknights and at the weekend by the age of 18. The fact that girls achieve autonomy before boys is not surprising given that girls reach puberty earlier than boys. Differences in behavioral autonomy between girls and boys have been demonstrated although this is heavily influenced by cultural factors (Bumpus et al., 2001). Girls have longer sleep time than boys from childhood (Franco et al., 2020) and sleep duration gradually decreases during teenage years (Thompson et al., 2024).

In our bivariate analysis, bedtime autonomy, both WA and TA, was associated with reduced sleep time, with insufficient sleep in the week and with catch up sleep at the weekend. However, this could be confounded by the known effects of age and sex on sleep duration. In our multivariate analysis adjusted for age, sex and factors known to be associated with time in bed (mood disorders, screen time and MEQ), we found potential sleep deprivation to be associated with both WA and, to a more marked extent TA, showing a dose response for increasing levels of autonomy. An association between autonomy and sleep duration has been found in older (Tashjian et al., 2019; Short et al., 2019) but not younger adolescents (Ewing et al., 2024; Meijer et al., 2016), and this may be due the progressive acquisition of weekend and weeknight autonomy with age and markedly reduced sleep time in the week in older adolescents with both weekend and weeknight autonomy.

Puberty leads to progressive delay in circadian rhythms (Carskadon, 1990). We observed an association between bedtime autonomy and evening preference (MEQr) probably mediated by age. In our multivariant analysis an association with reduced odds was found for the MEQ score and both potential sleep deprivation and social jetlag: higher scores, indicating morningness, are protective. Evening preference leads to later onset of sleep which in adolescents is potentially exacerbated by changes in the homeostatic system with a reduction in sleep pressure (Jenni et al., 2005). Increasing autonomy thus coincides with biological and behavioral changes that favor delayed sleep timing (Carskadon, 1990). When adolescents gain in autonomy and are given the opportunity to determine their own bedtime, these tendencies are likely to be expressed more fully, resulting in delayed sleep onset.

School starts between 08:00 and 08:30 in France, fixing a getting up time during the week which in our group was on average at 06:50 with reduced variability compared to weekend getting up times. This early getting up time, coupled with a later sleep onset time in adolescents who were free to fix their own bedtime leads to relative sleep restriction during the week in adolescents who need on average 9 h of sleep a night (Hirshkowitz et al., 2015). In our study, we found a significant reduction in weeknight time in bed of 1 h and 35 min in the TA group compared to the NA group. In the TA group, daytime sleepiness was significantly higher, implying that sleep duration was insufficient. We note that adolescents who were only autonomous at the weekend had longer time in bed in the week, implying that the reduction in time in bed in the week is driven by weeknight autonomy rather than autonomy *per se*. Our finding of reduced time in bed associated with autonomy is in line with Short's study using questionnaire data (Short et al., 2019) and Tashjian's study using actigraphy (Tashjian et al., 2019) but contrasts with (Meijer et al. 2016), who found that autonomy granting was not related to sleep variables but seemed to be more related to parental control and support. However, Meijers study was in a younger population aged 12–15 and our findings may be explained both by a wider age range but also by the distinction in our study between weeknight and weekend autonomy.

Sleep variability is emerging as an important determinant of health (Morales-Ghinaglia and Fernandez-Mendoza, 2023; Li et al., 2024) and academic performance (Wolfson and Carskadon, 1998). We only asked adolescents about their autonomy at bedtime, not whether they were autonomous for getting up times at the weekend. However, it is likely that adolescents with bedtime autonomy are also free to decide when they get up. This was supported by our finding that autonomous adolescents got up significantly later at the weekends. We defined social jetlag as a shift of more than 2 h in the midpoint of time in bed and this was slightly more common in adolescents with WA compared to those with TA. Our multivariate analysis confirmed that social jetlag was significantly associated with higher odds of WA but was not significantly associated with TA probably driven by the variability in bedtimes on weeknights vs. weekend. This confirms studies by Short et al. (2019) and by Illingworth in whom an increase in social jetlag of 31.2 min was found in adolescents with bedtime autonomy at the weekend (Illingworth et al., 2025). Our findings suggest that the relationship between sleep regularity and autonomy is less linear than that for time in bed. Partial autonomy thus contributes to misalignment between biological and social rhythms. Weekend autonomy is granted before weeknight autonomy and thus affects younger adolescents. Studies using functional brain imaging have shown that adolescence is a period of white matter maturation which progresses from the posterior to the anterior regions of the brain, with the uncinate fasciculus being one of the last tracts to be myelinated. This links the amygdala to the orbitofrontal and medial pre frontal cortex (Jamieson et al., 2020). Sleep variability has been shown to be a critical modifier of this maturational process, potentially impairing higher emotional and cognitive functioning (Baker et al., 2023).

In the present study, we assessed screen use in detail, with questions about time of screen use after dinner, once in bed and during the night. Screen use is highly prevalent in adolescents, increases with age and is associated with later bedtimes (Hartley et al., 2022; Varghese et al., 2021; Hale and Guan, 2015) due to the stimulating and phase shifting effects of light (Phipps-Nelson et al., 2003; West et al., 2011) and mental activity (Harbard et al., 2016). Defining excessive screen use is difficult: the American association of Pediatrics suggests a limit of 2 h a day, but as homework activities are often screen based this is regularly exceeded (Xu et al., 2019). Bivariate analysis showed an association between autonomy and excessive screen use in the evening, in bed and during the night: we note that the study by Tashjian did not find an association between screen use and autonomy but only looked at use before bed (Tashjian et al., 2019). In our multivariate analysis excessive screen use (more than 120 min in the evening) was strongly associated with higher odds of both potential sleep deprivation and with social jetlag, with use of screens in bed associated only with social jetlag. Use of screens in the evening and notably in bed potentially increases the physiological circadian phase shift and may contribute to reductions in sleep duration (Hartley et al., 2022): we note that screen use has been shown to be a mediator of the effects of autonomy on sleep duration (Tashjian et al., 2019).

In relation to daytime functioning, a number of studies have demonstrated that sleep variables (notably sleep duration, sleep timing and sleep quality) are associated with mood in adolescents (Wolfson and Carskadon, 1998; Widome et al., 2019; Tochigi et al., 2016). We did not find this in our multivariate analysis.

It has been demonstrated that increasing sleep duration and reducing sleep variability have a beneficial impact on adolescent health (Short et al., 2011). However, adolescents find themselves between a rock and a hard place. Getting up times in the week are fixed, and the only potential modifier is bedtime. In younger adolescents, parental intervention has been shown to be successful in advancing bedtime and increasing sleep duration (see Khor et al., 2021 for a review), but once autonomy has been granted, modifying sleep behavior depends on the adolescent themselves. Changing health behaviors is notoriously difficult and many theoretical models exist to explain this. Dual process models explain behavior change in terms of reflective/conscious and affective/impulsive systems (St Quinton and Brunton, 2017). In adolescents the latter systems, driven by the positive reward of social interactions and screen time at night and the aversive experience of early wakening are relatively strong compared to the reflective/conscious appreciation of the potential health benefits going to bed earlier. (Maskevich et al., 2022) showed that autonomously determined bedtime was regularly missed by adolescents, highlighting the difficulties of overcoming the affective/impulsive systems. Poor maturation of cognitive and emotional regulation driven by sleep variability in early teens (Baker et al., 2023) will further impair the ability to override immediate reward and positively modify behaviors. Bedtime autonomy presents clear risks for adolescent sleep duration and regularity. Prompting by parents has a clear role to reinforce the reflective/conscious systems, but our findings revealed that this was infrequently observed in both autonomous and non autonomous adolescents.

Our study has clear limitations; it is cross sectional and thus no conclusions about causality can be drawn. We relied on self report about bedtime autonomy and although we note a significant difference in bedtimes between the NA, WA and TA groups we have no information about how parents enforced bedtime rules in the NA and WA groups. We asked adolescents to self-report sleep timing and we calculated time in bed from mean bedtime and getting up times both during the week and at weekends. It is important to note that time in bed represents the total available window for sleep, rather than the actual sleep time. The latter will be reduced by the time taken to fall asleep and wake periods during the night. Our data is subjective, and objective data collection which allows an estimation of sleep duration across a prolonged period can only be done by actigraphy. Actigraphy in adolescents has shown correlation between objective and subjective sleep timing (Wolfson et al., 2003) although subjective sleep duration is typically overestimated (Lyall et al., 2020). We also relied on self-report for screen use which has been shown to be potentially inaccurate with a tendency to underestimation and does not capture multiple screen use (Araujo, 2017). Further studies should look at objective data from actigraphy, measures of screen use and consider pairing adolescent and parent questionnaires with diaries to examine day to day behaviors.

Our findings highlight the importance of bedtime autonomy in determining time in bed and sleep regularity. Decisions about when to grant bedtime autonomy should take into account age, school constraints and screen habits, and should be accompanied by targeted sleep education for both adolescents and parents aimed at preserving sleep duration and regularity. Health promotion programs aimed at improving adolescent sleep must consider autonomy as a key factor when designing effective interventions.

## Data Availability

The raw data supporting the conclusions of this article will be made available by the authors, without undue reservation.

## Glossary

NA non autonomous: the adolescent does not determine their bedtime.

WA weekend only autonomy: the adolescent decides when they go to bed at the weekend.

TA total autonomy: the adolescent decides when they go to bed at the weekend and on weeknights.

## References

[B1] Araujo T. (2017). Communication methods and measures how much time do you spend online? Understanding and improving the accuracy of self-reported measures of internet use. Taylor Francis 11, 173–190. doi: 10.1080/19312458.2017.1317337

[B2] Baker A. E. Tashjian S. M. Goldenberg D. Galván A. (2023). Sleep variability over a 2-week period is associated with restfulness and intrinsic limbic network connectivity in adolescents. Sleep 46:zsac248. doi: 10.1093/sleep/zsac248 36223429 PMC9905777

[B3] Bocéréan C. Dupret E. (2014). A validation study of the hospital anxiety and depression scale (HADS) in a large sample of French employees. BMC Psychiatry 14:354. doi: 10.1186/s12888-014-0354-0 25511175 PMC4476305

[B4] Bumpus M. F. Crouter A. C. McHale S. M. (2001). Parental autonomy granting during adolescence: exploring gender differences in context. Dev. Psychol. 37, 163–173. doi: 10.1037/0012-1649.37.2.163 11269385

[B5] Caci H. Deschaux O. Adan A. Natale V. (2009). Comparing three morningness scales: age and gender effects, structure and cut-off criteria. Sleep Med. 10, 240–245. doi: 10.1016/j.sleep.2008.01.007 18387342

[B6] Carskadon M. A. (1982). “The second decade,” in Sleep and Waking Disorders: Indications and Techniques, ed. C. Guilleminaut (Menlo Park, CA: Addison Wesley), 99–125.

[B7] Carskadon M. A. (1990). Patterns of sleep and sleepiness in adolescents. Pediatrician 17, 5–12. 2315238

[B8] Carskadon M. A. (2011). Sleep in adolescents: the perfect storm. Pediatr. Clin. North Am. 58, 637–647. doi: 10.1016/j.pcl.2011.03.003 21600346 PMC3130594

[B9] de Souza C. M. Hidalgo M. P. L. (2014). Midpoint of sleep on school days is associated with depression among adolescents. Chronobiol. Int. 31, 199–205. doi: 10.3109/07420528.2013.838575 24156519

[B10] Ewing E. L. Xia M. Gunn H. E. (2024). Affiliative parent-adolescent bedtime and waketime interactions are associated with adolescent sleep. Behav. Sleep Med. 22, 168–178. doi: 10.1080/15402002.2023.2217970 37318033 PMC10721726

[B11] Franco P. Putois B. Guyon A. Raoux A. Papadopoulou M. Guignard-Perret A. . (2020). Sleep during development: sex and gender differences. Sleep Med. Rev. 51:101276. doi: 10.1016/j.smrv.2020.101276 32109833

[B12] Gunn H. E. O'Rourke F. Dahl R. E. Goldstein T. R. Rofey D. L. Forbes E. E. . (2019). Young adolescent sleep is associated with parental monitoring. Sleep Health 5, 58–63. doi: 10.1016/j.sleh.2018.09.001 30670167 PMC6347380

[B13] Gustin M.- P. Putois B. Guyon A. Lecendreux M. Challamel M.- J. Plancoulaine S. . (2023). French sleepiness scale for adolescents-8 items: a discriminant and diagnostic validation. Encephale 49, 109–116. doi: 10.1016/j.encep.2022.06.004 36253180

[B14] Hale L. Guan S. (2015). Screen time and sleep among school-aged children and adolescents: a systematic literature review. Sleep Med. Rev. 21, 50–58. doi: 10.1016/j.smrv.2014.07.007 25193149 PMC4437561

[B15] Hansen M. Janssen I. Schiff A. Zee P. C. Dubocovich M. L. (2005). The impact of school daily schedule on adolescent sleep. Pediatrics 115, 1555–1561. doi: 10.1542/peds.2004-1649 15930216

[B16] Harbard E. Allen N. B. Trinder J. Bei B. (2016). What's keeping teenagers up? Prebedtime behaviors and actigraphy-assessed sleep over school and vacation. J. Adolesc. Health 58, 426–32. doi: 10.1016/j.jadohealth.2015.12.011 26874590

[B17] Hartley S. Royant-Parola S. Zayoud A. Gremy I. Matulonga B. (2022). Do both timing and duration of screen use affect sleep patterns in adolescents? PLoS ONE 17:e0276226. doi: 10.1371/journal.pone.0276226 36264928 PMC9584513

[B18] Hirshkowitz M. Whiton K. Albert S. M. Alessi C. Bruni O. DonCarlos L. . (2015). National Sleep Foundation's sleep time duration recommendations: methodology and results summary. Sleep Health 1, 40–43. doi: 10.1016/j.sleh.2014.12.010 29073412

[B19] Illingworth G. Manchanda T. Skripkauskaite S. Fazel M. Waite F. (2025). Social jetlag and sleep habits in children and adolescents: associations with autonomy (bedtime setting and electronics curfew) and electronic media use before sleep. Chronobiol. Int. 42, 46–57. doi: 10.1080/07420528.2024.2444675 39760865 PMC11854036

[B20] Jamieson D. Broadhouse K. M. Lagopoulos J. Hermens D. F. (2020). Investigating the links between adolescent sleep deprivation, fronto-limbic connectivity and the onset of mental disorders: a review of the literature. Sleep Med. 66, 61–67. doi: 10.1016/j.sleep.2019.08.013 31791002

[B21] Jenni O. G. Achermann P. Carskadon M. A. (2005). Homeostatic sleep regulation in adolescents. Sleep 28, 1446–1454. doi: 10.1093/sleep/28.11.1446 16335485

[B22] Khor S. P. H. McClure A. Aldridge G. Bei B. Yap M. B. H. (2021). Modifiable parental factors in adolescent sleep: a systematic review and meta-analysis. Sleep Med. Rev. 56:101408. doi: 10.1016/j.smrv.2020.101408 33326915

[B23] Li M. Zhang Y. Huang M. Fan Y. Wang D. Ma Z. . (2024). Prevalence, correlates, and mental health outcomes of social jetlag in Chinese school-age adolescents: a large-scale population-based study. Sleep Med. 119, 424–431. doi: 10.1016/j.sleep.2024.05.039 38781665

[B24] Lohaus A. (2018). Developmental Psychology of Adolescence. Berlin: Springer.

[B25] Lyall L. M. Sangha N. Wyse C. Hindle E. Haughton D. Campbell K. . (2020). Accelerometry-assessed sleep duration and timing in late childhood and adolescence in Scottish schoolchildren: a feasibility study. PLoS ONE 15:e0242080. doi: 10.1371/journal.pone.0242080 33259503 PMC7707491

[B26] Maskevich S. Shen L. Drummond S. P. A. Bei B. (2022). What time do you plan to sleep tonight? An intense longitudinal study of adolescent daily sleep self-regulation via planning and its associations with sleep opportunity. J. Child Psychol. Psychiatry 63, 900–911. doi: 10.1111/jcpp.13540 34811748

[B27] Meijer A. M. Reitz E. Dekovic M. (2016). Parenting matters: a longitudinal study into parenting and adolescent sleep. J. Sleep Res. 25, 556–564. doi: 10.1111/jsr.12406 27178659

[B28] Morales-Ghinaglia N. Fernandez-Mendoza J. (2023). Sleep variability and regularity as contributors to obesity and cardiometabolic health in adolescence. Obes. Silver Spring. Md. 31, 597–614. doi: 10.1002/oby.23667 PMC9975080 36754840

[B29] Phipps-Nelson J. Redman J. R. Dijk D. J. Rajaratnam S. M. W. (2003). Daytime exposure to bright light, as compared to dim light, decreases sleepiness and improves psychomotor vigilance performance. Sleep 26, 695–700. doi: 10.1093/sleep/26.6.695 14572122

[B30] Pu Z. Leong R. L. F. Chee M. W. L. Massar S. A. A. (2022). Bedtime procrastination and chronotype differentially predict adolescent sleep on school nights and non-school nights. Sleep Health 8, 640–647. doi: 10.1016/j.sleh.2022.09.007 36272919

[B31] Short M. A. Gradisar M. Lack L. C. Wright H. R. Chatburn A. (2013). Estimating adolescent sleep patterns: parent reports versus adolescent self-report surveys, sleep diaries, and actigraphy. Nat. Sci. Sleep 5, 23–6. doi: 10.2147/NSS.S38369 23620690 PMC3630985

[B32] Short M. A. Gradisar M. Wright H. Lack L. C. Dohnt H. Carskadon M. A. . (2011). Time for bed: parent-set bedtimes associated with improved sleep and daytime functioning in adolescents. Sleep 34, 797–800. doi: 10.5665/SLEEP.1052 21629368 PMC3098947

[B33] Short M. A. Kuula L. Gradisar M. Pesonen A.- K. (2019). How internal and external cues for bedtime affect sleep and adaptive functioning in adolescents. Sleep Med. 59, 1–6. doi: 10.1016/j.sleep.2018.11.018 31150946

[B34] St Quinton T. Brunton J. A. (2017). Implicit processes, self-regulation, and interventions for behavior change. Front. Psychol. 8:346. doi: 10.3389/fpsyg.2017.00346 28337164 PMC5340749

[B35] Tashjian S. M. Mullins J. L. Galván A. (2019). Bedtime autonomy and cellphone use influence sleep duration in adolescents. J. Adolesc. Health 64, 124–130. doi: 10.1016/j.jadohealth.2018.07.018 30366713

[B36] Thompson M. J. Gillis B. T. Hinnant J. B. Erath S. A. Buckhalt J. A. El-Sheikh M. . (2024). Trajectories of actigraphy-derived sleep duration, quality, and variability from childhood to adolescence: downstream effects on mental health. Sleep 47:zsae112. doi: 10.1093/sleep/zsae112 38758702 PMC11321856

[B37] Tochigi M. Usami S. Matamura M. Kitagawa Y. Fukushima M. Yonehara H. . (2016). Annual longitudinal survey at up to five time points reveals reciprocal effects of bedtime delay and depression/anxiety in adolescents. Sleep Med. 17, 81–86. doi: 10.1016/j.sleep.2015.08.024 26847979

[B38] Varghese N. E. Santoro E. Lugo A. Madrid-Valero J. J. Ghislandi S. Torbica A. . (2021). The role of technology and social media use in sleep-onset difficulties among italian adolescents: cross-sectional study. J. Med. Internet Res. 23:e20319. doi: 10.2196/20319 33475517 PMC7862002

[B39] West K. E. Jablonski M. R. Warfield B. Cecil K. S. James M. Ayers M. A. . (2011). Blue light from light-emitting diodes elicits a dose-dependent suppression of melatonin in humans. J. Appl. Physiol. 110, 619–626. doi: 10.1152/japplphysiol.01413.2009 21164152

[B40] White D. Leach C. Sims R. Atkinson M. Cottrell D. (1999). Validation of the hospital anxiety and depression scale for use with adolescents. Br. J. Psychiatry J. Ment. Sci. 175, 452–454. doi: 10.1192/bjp.175.5.452 10789277

[B41] Widome R. Berger A. T. Lenk K. M. Erickson D. J. Laska M. N. Iber C. . (2019). Correlates of short sleep duration among adolescents. J. Adolesc. 77, 163–167. doi: 10.1016/j.adolescence.2019.10.011 31739274 PMC7015268

[B42] Wittmann M. Dinich J. Merrow M. Roenneberg T. (2006). Social jetlag: misalignment of biological and social time. Chronobiol. Int. 23, 497–509. doi: 10.1080/07420520500545979 16687322

[B43] Wolfson A. R. Carskadon M. A. (1998). Sleep schedules and daytime functioning in adolescents. Child Dev. 69, 875–887. doi: 10.1111/j.1467-8624.1998.tb06149.x 9768476

[B44] Wolfson A. R. Carskadon M. A. Acebo C. Seifer R. Fallone G. Labyak S. E. . (2003). Evidence for the validity of a sleep habits survey for adolescents. Sleep 26, 213–216. doi: 10.1093/sleep/26.2.213 12683482

[B45] Xu F. Adams S. K. Cohen S. A. Earp J. E. Greaney M. L. (2019). Relationship between physical activity, screen time, and sleep quantity and quality in US adolescents aged 16–19. Int. J. Environ. Res. Public Health 16:1524. doi: 10.3390/ijerph16091524 31052159 PMC6539318

