# Supplementary material for: The association between perceived bedtime autonomy, sleep patterns, and daytime functioning in adolescents
Source: Front Sleep. 2026 May 25;5:1719668. doi: 10.3389/frsle.2026.1719668 (PMC13243058; doi:10.3389/frsle.2026.1719668)
Supplement: Supplementary file 1 [file Table_1.docx]

Supplementary table 1 : questions about screen use

| Do you have any of the following in your bedroom (multiple responses possible) : television, games console, computer, tablet |
| --- |
| Do you keep your smartphone in your bedroom overnight? (Yes/no) |
| If Yes is it in flight mode, in do not disturb mode, with the alerts desactivated, active all night |
| What do you do in the evening after dinner? (multiple responses possible): I work (including on a screen), I watch the television, videos or series, I listen to music, I read, I play video games, I use social media, I talk with friends via a messaging service |
| How much time do you spend on a screen after dinner : less than 1 hour, 1-2 hours, 2-3 hours more than 3 hours |
| What do you do most often once you are in bed: nothing I try to sleep; I watch the television or videos on my screen, I listen to music, I read, I play video games, I use social media, I talk with friends via a messaging service |
| Do you wake up regularly (more that 3 times a week) during the night to spend time on your screen? |
| If yes do you: wake up spontaneously, are woken by an alert, programme an alarm on your device to wake you up |
| How much time do you spend on your screen during the night? Less than 30 minutes, 30-60 minutes 1-2 hours, more than 2 hours |
| During your screen time in the night what do you mostly do? Social media, watch videos, play video games, a mixture of the above |
